# Experimental Investigation on Cutting Characteristics in Nanometric Plunge-Cutting of BK7 and Fused Silica Glasses

**DOI:** 10.3390/ma8041428

**Published:** 2015-03-27

**Authors:** Qinglong An, Weiwei Ming, Ming Chen

**Affiliations:** School of Mechanical Engineering, Shanghai Jiao Tong University, Shanghai 200240, China; E-Mails: qlan@sjtu.edu.cn (Q.A.); mingseas@sjtu.edu.cn (W.M.)

**Keywords:** nanometric cutting, ductile-brittle cutting, CUCT, machined surface morphology, size effect, specific cutting energy

## Abstract

Ductile cutting are most widely used in fabricating high-quality optical glass components to achieve crack-free surfaces. For ultra-precision machining of brittle glass materials, critical undeformed chip thickness (CUCT) commonly plays a pivotal role in determining the transition point from ductile cutting to brittle cutting. In this research, cutting characteristics in nanometric cutting of BK7 and fused silica glasses, including machined surface morphology, surface roughness, cutting force and specific cutting energy, were investigated with nanometric plunge-cutting experiments. The same cutting speed of 300 mm/min was used in the experiments with single-crystal diamond tool. CUCT was determined according to the mentioned cutting characteristics. The results revealed that 320 nm was found as the CUCT in BK7 cutting and 50 nm was determined as the size effect of undeformed chip thickness. A high-quality machined surface could be obtained with the undeformed chip thickness between 50 and 320 nm at ductile cutting stage. Moreover, no CUCT was identified in fused silica cutting with the current cutting conditions, and brittle-fracture mechanism was confirmed as the predominant chip-separation mode throughout the nanometric cutting operation.

## 1. Introduction

Precision optical components, made of hard-brittle materials such as ceramics and glasses, always require high-quality surface with good edge and crack-free zone. However, fabricating these components still is the most difficult and challenging task for modern manufacturing sectors due to the key obstacle to achieve acceptable machining quality. In spite of this, the demands for microfeatures in these materials have been increasing for a number of miniature-product applications such as MEMS (Micro-Electro-Mechanical System) device packaging, mini-vision systems and microelectronic packaging [1]. Unfortunately, the hard and brittle features of glasses easily result in micro-cracks or rough surface during conventional machining process [2,3,4,5]. With the development of advanced manufacturing technology, ultra-precision cutting using single-crystal diamond (SCD) tools has potentially become a feasible solution for manufacturing these high-quality optical components with submicrometric and nanometric accuracy, and nanometric surface roughness [6,7].

Boroscilate crown glass (BK7) and silicon dioxide glass (bulk SiO2, fused silica) are two common optical materials for fabricating of high-quality optical components due to their excellent properties, such as good scratch resistance, bubble-free, and high optical transmission in the visible range [8,9]. The mechanical properties of BK7 and fused silica are shown in Table 1. These hard and brittle characteristics would easily cause micro-fractures and micro-cracks during ultra-precision cutting process. It has been investigated that there always exists a critical undeformed chip thickness (CUCT) in ductile cutting, below which high surface finish with crack-free surface can be achieved [10]. In the past few decades, numerous studies concerning ductile-brittle transition process have been conducted to clarify the mechanism of hard-brittle material machining. Blake and Scattergood [11] studied the CUCT for single crystals of germanium and silicon machining by using the diamond-turning lathe, and proposed several optimal cutting parameters for the ductile cutting, such as CUCT, tool geometries, and cutting speed. Ravindra and Patten [12] investigated the ductile regime of fused silica via single point diamond turning and found that the strength, hardness and fracture toughness of workpiece material played as the key factors that significantly affected the extent of brittle fracture. Zhou *et al.* [13] investigated the brittle-ductile transition issue with diamond cutting of silicon from the viewpoint of material response and tool geometry. It was found that tool rake angle would greatly influence the brittle-ductile transition. Liu *et al*. [14] carried out nanometric cutting trials to evaluate the cutting performance of tungsten carbide material in ductile mode by using Cubic Boron Nitride (CBN) cutters, and obtained the CUCT at the ductile-brittle transition point. Moreover, Arif *et al.* [15] established a predictive model regarding CUCT estimation for ductile-brittle transition, and validated the applicability in machining of single crystal silicon and BK7 glass. These studies have shown that CUCT exhibited consistent relation with the nature of work materials and machining conditions including cutting parameters and tool geometries. To determine the ductile-brittle transition point, high-precision machine tools and diamond cutting tools with nanometric precision are urgently required. It is of interest to characterize the CUCT and material removal regime of different hard-brittle materials by nanometric plunge-cutting experiments.

In this study, nanometric plunge-cutting experiments were performed on an ultra-high-precision CNC machining center. Cutting characteristics were investigated for both BK7 and fused silica according to the variation of surface texture generation with increasing undeformed chip thickness. In addition, several aspects including surface roughness, cutting-force generation and specific cutting energy were precisely investigated aiming to clarify the ductile-brittle transition point and the material removal regime during ductile and brittle cutting.

**Table 1:** Mechanical properties of boroscilate crown glass (BK7) and fused silica [16,17,18].

Materials	BK7	Fused silica
Mode I, Fracture toughness *K*_IC_ (MPa·m^1/2^)	0.82	0.75
Young’s modulus *E* (GPa)	81	71.5
Hardness *H* (GPa)	5.8	9.22

## 2. Experimental Section

**Figure 1:**
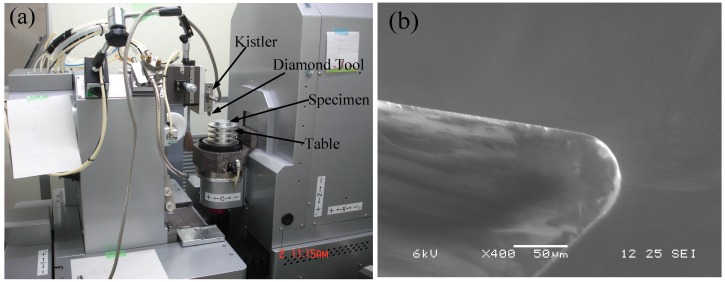
(**a**) Experimental setup on Ultra Nano 100; (**b**) SEM (Scanning electron microscope) morphology of the single-crystal diamond (SCD) tool.

**Figure 2:**
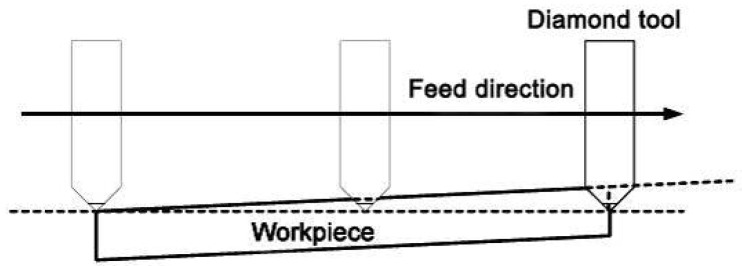
Schematic diagram of plunge-cutting operation.

Plunge-cutting experiments were carried out on a 5-axis ultra-high-precision CNC (computer numerical control) machining center (Ultra Nano 100, Sodick Corporation, Yokohama, Japan) with a position detection and motion control by minimum unit of 0.07 nm, as shown in Figure 1a. Single-crystal diamond (SCD) tool was used with nose radius of 50 μm, cutting edge radius of 20 nm, rake angle of 0°, clearance angle of 7° and orientation (110°) surface as the rake face. Figure 1b shows the SCD tool prepared with good integrity on the cutting edge and surface roughness of 9 nm on the rake face. BK7 and fused silica samples with a dimension of 10 mm × 10 mm × 5 mm were mechanically polished with surface roughness below 0.8 nm. In the plunge-cutting experiments [19,20], the samples were fixed on a vacuum-floated table that was slightly tilted on one side, as shown in Figure 2. The maximum depths of cut were set as 2500 nm and 4750 nm for BK7 and fused silica respectively. The varying undeformed chip thickness will help to find the ductile-brittle transition and investigate on the material removal regime of BK7 and fused silica, such as plowing, ductile removal and brittle removal [21,22]. A constant cutting speed of 300 mm/min was used throughout the plunge-cutting experiments.

Cutting forces were measured by a piezoelectric dynamometer (model 9256C1, Kistler Corporation, Winterthur, Switzerland). The onset of brittle fracture was carefully observed through 3D surface morphologies and cross-sections of the groove, which were detected by an optical profilometry (New View 5032, Zygo Corporation, Middlefield, CT, USA) with MetroPro^®^ software. The optical profilometry was also used to measure the nanometric surface roughness along the bottom of cutting groove in the feed direction. Scanning electron microscope (SEM) (model JSM-5600LV, JEOL Corporation, Tokyo, Japan) was also used to characterize the machined surface texture, such as fractures and micro-chips. 

## 3. Machining Regime of Brittle Materials

**Figure 3:**
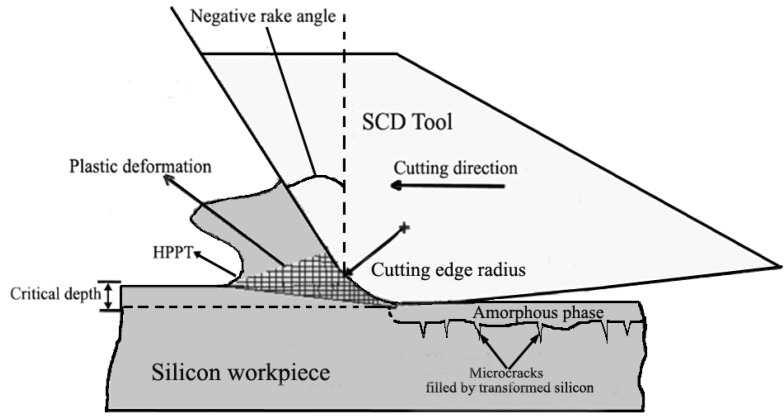
Ductile regime machining of brittle materials.

**Figure 4:**
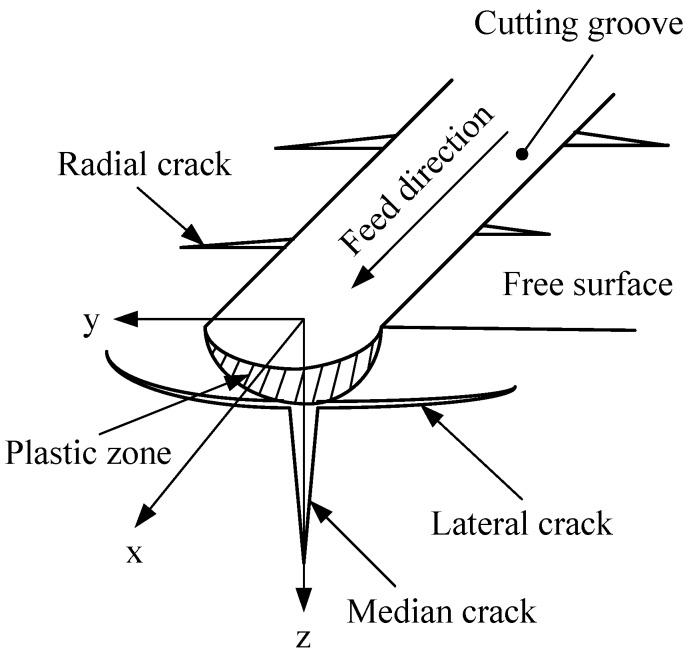
Schematic of cracking induced by plunge-cutting of brittle materials.

As shown in Figure 2, the test specimen experiences elastic deformation, scratching and plowing successively during the plunge-cutting operation. With increasing of undeformed chip thickness, it will lead to larger cutting force, which will consequently result in higher compressive stress concentrated in the tool-workpiece area. When the compressive stress exceeds the fracture limit, cracks and brittle fracture tend to arise and propagate. Ravindra and Patten [12] revealed that sufficient compressive stress would cause a ductile mode behavior, in which the material was removed by plastic deformation instead of brittle fracture. This micro-scale phenomenon exhibited strong relation to High Pressure Phase Transformation (HPPT) of the work material [12,23,24,25]. Figure 3 shows the schematic of ductile regime machining of brittle materials. It depicts a graphical representation of the highly stressed zone that results in ductile machining. It is noticeable that negative rake angle shows beneficial effects on ductile-cutting performance. It also shows a crack self-healing mechanism on the machined surface during ductile cutting of silicon. It has been revealed with transmission electron microscope (TEM) by Kovalchenko *et al*. [26]. With regard to ductile-brittle transition, Ahn *et al*. [27] proposed three crack patterns (radial crack, lateral crack and median crack), which may exist during nano or micro plunge-cutting of brittle materials, as illustrated in Figure 4. It was assumed that the tensile stress occurred in the elastic zone initiates and propagates the crack patterns. Cracking will be motivated from the plastic/elastic interface with high residual stress. The load exerted on the contact zone was used to determine the occurrence of these crack patterns [28]. There are also many cracks in front of the cutting tool, which will be removed with the chip separation.

### 3.1. Specific Cutting Energy

**Figure 5:**
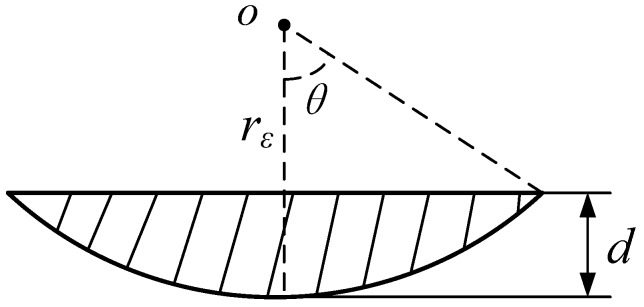
Cross section of the plunge-cut groove.

Specific cutting energy represents the energy consumed in removing a unit volume of materials. It exhibits strong relation with the cutting load and also can be used to distinguish the mode of material removal [29]. The specific cutting energy is expressed as:

(1) $$E_{1}=\frac{F_{c}}{A}$$

where *F_c_* is the feed cutting force, and *A* is the cross-sectional area of the groove, as shown in Figure 5. *A* can be calculated as:

(2) $$A={r_{\text{ε}}}^{2}\arccos\frac{r_{\text{ε}}-d}{r_{\text{ε}}}-(r_{\text{ε}}-d)\sqrt{2r_{\text{ε}}d-d^{2}}$$

where *r*_ε_ is the nose radius of the SCD tool, and *d* is the depth of the groove (also as undeformed chip thickness).

### 3.2. Critical Undeformed Chip Thickness

The cutting force and cross-sectional area both depend significantly on the undeformed chip thickness. It is believed that there always exists a critical value when an initial crack appears at the ductile-brittle transition point, which can be precisely observed by Zygo optical profilometry in this study. Based on Griffith fracture propagation criterion, Bifano *et al*. [10] proposed the model of CUCT based on the continuum mechanics as:

(3) $$d_{c}=\text{β}\frac{E}{H}{\left(\frac{K_{\text{IC}}}{H}\right)}^{2}$$

where *d_c_* is CUCT, *E* is the Young’s modulus, *H* is the nano-hardness, *K*_IC_ is the fracture toughness of mode I, and β is a dimensionless material constant determined by the cutting conditions. β was estimated as 0.15 for brittle materials derived by Bifano [10] in precision grinding tests. According to the mechanical properties of BK7 and fused silica as described in Table 1, the CUCT for BK7 and fused silica could be calculated as 42 nm and 8 nm for BK7 and fused silica, respectively. In this study, nanometric plunge-cutting experiments would be performed to study the material removal regime of BK7 and fused silica by carefully observation of CUCT, which would also be used to make a comparative analysis with the calculated values.

## 4. Results and Discussion

### 4.1. Machined Surface Morphology

**Figure 6:**
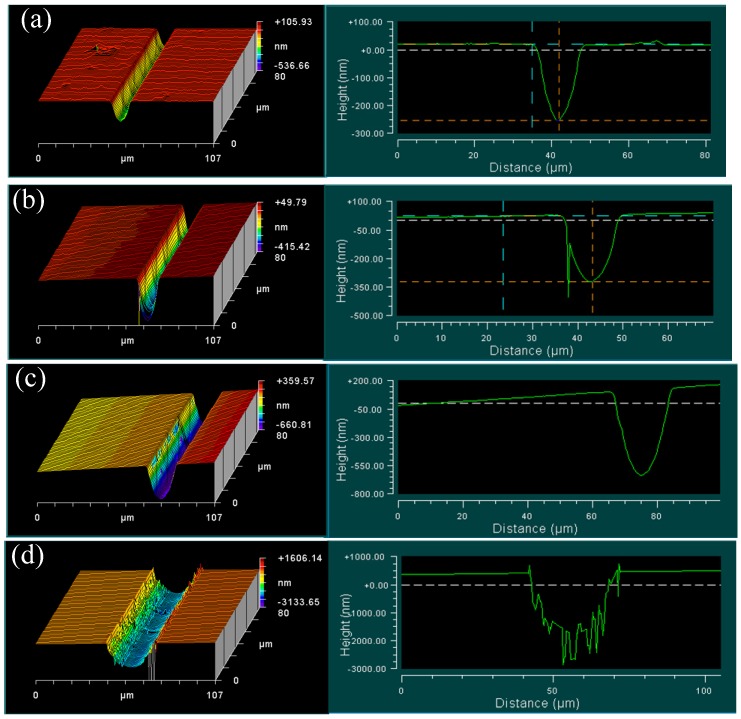
3D surface morphologies and cross-sections of cutting groove at different undeformed chip thickness for BK7: (**a**) 272 nm; (**b**) 320 nm; (**c**) 800 nm; (**d**) 2100 nm.

The measured 3D surface morphologies and cross-sections of the cutting groove for BK7 are shown in Figure 6. In these pictures, a smooth curve of cross-section with no intermittence represents the free-occurrence of crack defects on the machined surface. It is noticeable that there were no cracks on the machined surface until the first crack appeared on one side of the groove with undeformed chip thickness as 320 nm, as shown in Figure 6a,b. The crack proves the occurrence of brittle fracture in the cutting process. This stage was defined as ductile cutting and CUCT could be determined as 320 nm, which is almost the same with Muhammad’ result [15]. With the increased undeformed chip thickness, more cracks appeared randomly on the side or edge of the groove. Sometimes it showed a crack-free machined surface, as shown in Figure 6c. This stage was defined as ductile-brittle transition cutting. When the undeformed chip thickness approached to 2100 nm, many cracks in large-size dimension appeared near the bottom of the groove. These cracks were caused by the brittle fracture of bulk material, and were harmful for surface integrity. 2100 nm could be determined as the outset of the brittle cutting.

**Figure 7:**
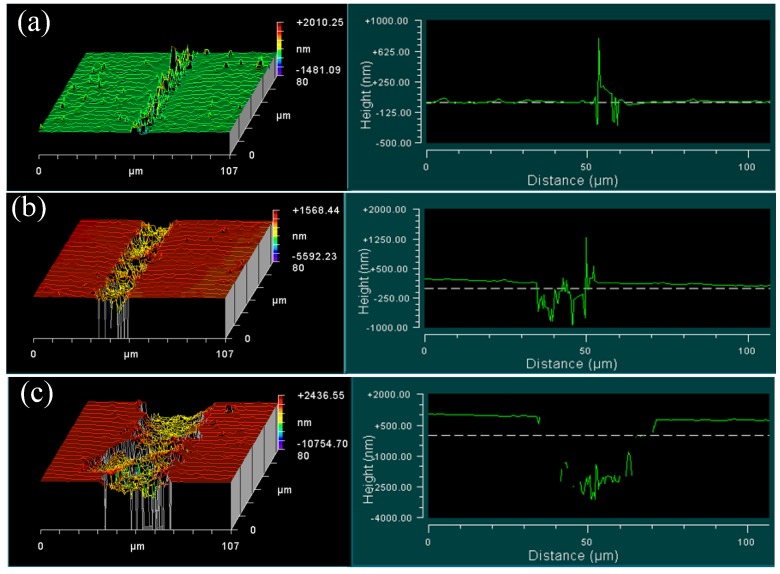
3D surface morphology and cross-section of cutting groove for fused silica: (**a**) 30 nm; (**b**) 307 nm; (**c**) 2895 nm.

Figure 7 shows the 3D surface morphologies and cross-sections of cutting groove with different undeformed chip thickness for fused silica glass. It can be observed that fused silica cutting was characterized by a typical brittle-regime with numerous fractures. There were many projections on the cutting trace even with the undeformed chip thickness as 30 nm, as shown in Figure 7a. These projections are pileups of debris principally caused by the squeezing effects of the SCD tool. With further increase of the undeformed chip thickness, a large amount of irregular fractures, concavities and projections appeared throughout the cutting groove [30], as shown in Figure 7b,c. In the plunge-cutting of fused silica, no CUCT was found due to the limitation of observation condition. The CUCT for fused silica would be estimated below 30 nm. A SCD tool with a negative rake angle would help get CUCT [12].

**Figure 8:**
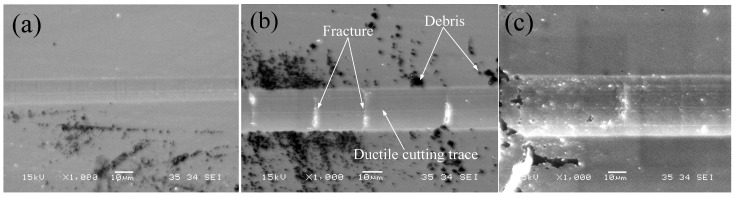
SEM photograph of cutting groove topography for BK7 at 300 mm/min: (**a**) ductile cutting; (**b**) ductile-brittle transition; (**c**) brittle cutting.

**Figure 9:**
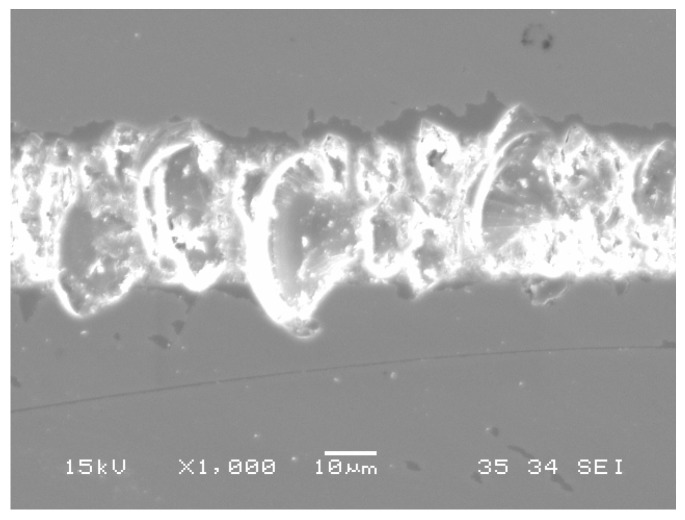
SEM micrographs of cutting grooves for fused silica glass.

As presented above, BK7 and fused silica show distinct removal regimes in the nanometric plunge-cutting process. Typically three stages of ductile, ductile-brittle transition and brittle could be observed for BK7 cutting, while the fused silica cutting only exhibited the brittle stage with numerous brittle fractures of materials. Such phenomena can also be confirmed from the following SEM micrographs of the cutting grooves, as shown in Figure 8 and Figure 9.

At the beginning of ductile cutting, there are many sliding and plowing traces on the machined surface of the cutting groove, as shown in Figure 8a. In the period of the ductile-brittle transition, smooth ductile cutting traces and stochastic emergence of lateral and median cracks coexisted on the machined surface of the groove, as shown in Figure 8b. In addition, there was a lot of debris piling up on both sides of cutting groove. The lateral and median cracks commonly led to the fractures on the surface of the cutting groove. With increased undeformed chip thickness, massive continuous cracks were promoted on both sides and bottom of the cutting groove during the brittle cutting, as shown in Figure 8c. Furthermore, the experimental results also validated the surface generation process of BK7 cutting, as illustrated in Figure 4.

Figure 9 shows the massive fractures of fused silica after plunge-cutting. It can be seen that these fractures caused an incomplete and irregular groove with rough profiles and surfaces. It shows that brittle cutting was the main material removal mode in fused silica cutting, which exhibited significant disparity with BK7 cutting. There existed many cracks but few fractures during BK7 brittle-cutting stage, which was caused by its low hardness and high fracture toughness. Some researchers have found that a SCD tool with a negative rake angle and smaller undeformed chip thickness would promote ductile behavior and help get a smooth machined surface [12]. Heating (laser assisted machining), vibration or chemically assisted methods would also help get a good machining quality in fused silica machining [31,32].

As presented above, CUCT of BK7 was observed as 320 nm and no CUCT was found for fused silica in the plunge-cut tests. The experimental results are different from the theoretical CUCT calculated with Equation (3) in Section 3.2. The reason can be attributed to the cutting conditions, such as cutting speed and tool geometry. In Bifano’s tests, high cutting speed (almost 1000 times as this research) and blunt abrasive grain tool were used. These cutting conditions would determine the dimensionless material constant β, as the value of hardness (nano or micro) does.

### 4.2. Surface Roughness

**Figure 10:**
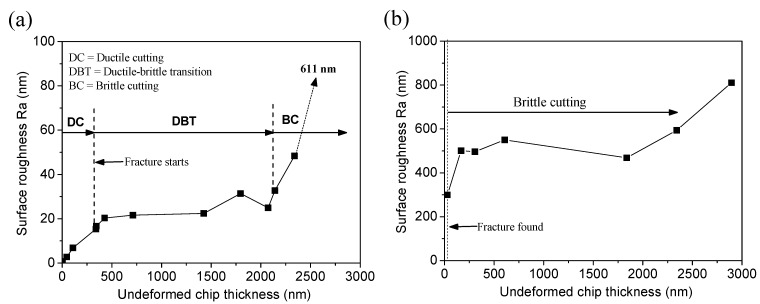
Variation of surface roughness with undeformed chip thickness: (**a**) BK7; (**b**) Fused silica.

Surface finish of the cutting groove was investigated to characterize the surface generation regime. Surface roughness was measured along the bottom of groove. Low surface roughness value always signifies a smooth surface with minimum cracks or fractures. Figure 10a shows the variation of surface roughness with the undeformed chip thickness for BK7. It can be seen that there existed two turning points on the surface roughness curve, where BK7 experienced ductile-brittle transition and brittle cutting. The surface roughness value was kept lower than 20 nm in ductile cutting stage with undeformed chip thickness below 320 nm. In ductile-brittle transition cutting stage, with undeformed chip thickness from 320 to 2100 nm, the surface roughness values were always kept within the range of 20 to 30 nm. When entering brittle cutting stage, surface roughness value suffered a dramatic increase as shown in Figure 10a. The high surface roughness value was attributed to the large amount of cracks and fractures formed on the machined surface. As shown in Figure 10b, the surface roughness of fused silica groove always maintained at a high value within the range from 300 to 800 nm. It was attributed to the fact that brittle cutting gave rise to the high surface roughness value.

### 4.3. Cutting Force and Specific Cutting Energy

**Figure 11:**
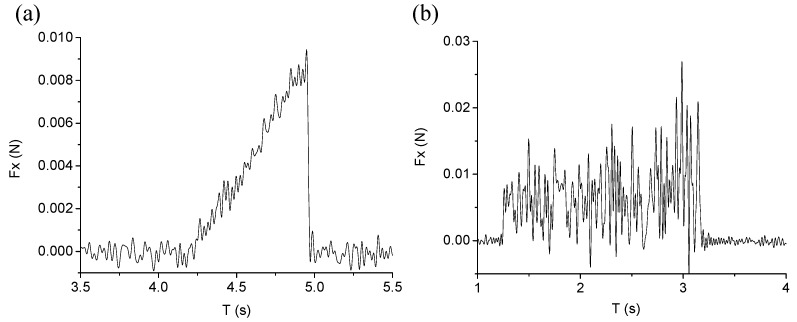
Variation of feed cutting force with cutting speed of 300 mm/min: (**a**) BK7; (**b**) Fused silica.

Figure 11 shows the variation of feed cutting force during the plunge-cutting experiments. It can be seen that the feed cutting forces for BK7 and fused silica show different characteristics. The feed cutting force mostly increased linearly with increasing undeformed chip thickness for BK7, while the feed cutting force for fused silica cutting fluctuated violently and the amplitude of fluctuation also increased when undeformed chip thickness increased. The maximum feed cutting force for fused silica was nearly three times as that for BK7. The mechanism controlling such physical phenomena can be attributed to the different material removal modes dominating for BK7 and fused silica cutting. Although there existed ductile-brittle transition cutting and brittle cutting, ductile cutting is always identified as the key material removal mode during BK7 cutting process. In addition, the difference can also be explained by the topographies of cutting groove, as shown in Figure 8. For ductile cutting, the feed cutting force increased with increasing undeformed chip thickness. While for fused silica, brittle cutting was the main material removal mode, and the whole cutting process was dominated by brittle fractures. The phenomena can also be seen from the topography of cutting groove, as shown in Figure 9. Large brittle fractures led to the larger cutting force and fluctuation during fused silica cutting process.

**Figure 12:**
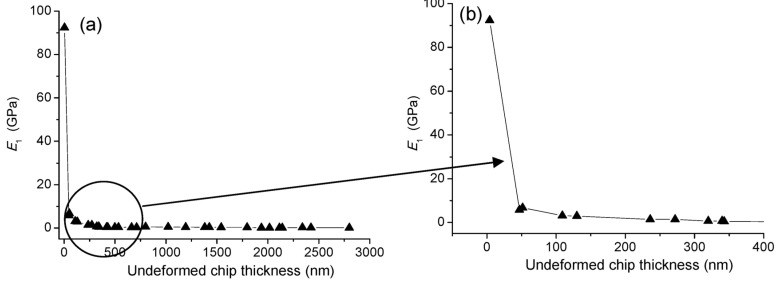
(**a**) Specific cutting energy for BK7 with cutting speed of 300 mm/min; (**b**) zoom-in view.

The material removal mechanism for BK7 cutting could also be revealed by specific cutting energy. As shown in Figure 12, the specific cutting energy for BK7 cutting had a high value above 80 GPa at the beginning of plunge cutting. With increasing of undeformed chip thickness, the specific cutting energy decreased quickly, which could be due to the size effect of the deformation zone with smaller undeformed chip thickness [29]. In ductile cutting stage, especially with undeformed chip thickness below 320 nm, BK7 specimen would experience a typical plastic deformation and size effects of cutting edge. It can be seen from Figure 12 that there is a turning point at the undeformed chip thickness of 50 nm or so. The specific cutting energy decreased quickly from 92 to 6 GPa within the range of undeformed chip thickness below 50 nm. It is because the sliding and plowing actions of the cutting tool occupied the primary cutting operation at the beginning of plunge-cutting test. When undeformed chip thickness was larger than 50 nm, the specific cutting energy decreased slowly, which means that the shearing of the SCD tool became the predominant material removal mode in the cutting process. So 50 nm could be defined as the size effect CUCT in the plunge-cutting test. Therefore, to get a superior surface integrity in BK7 precision machining, undeformed chip thickness should be selected between 50 and 320 nm when cutting with the SCD tool and cutting speed of 300 mm/min. As shown in Figure 12, the specific cutting energy almost kept constant during ductile-brittle transition and brittle cutting stages. It means that no additional energy was consumed when the undeformed chip thickness was greater than the critical value. It was because, surface energy of the material kept constant and hence equal amount of energy was required to break the surface bonds with undeformed chip thickness greater than the critical value [15].

It was different from BK7 cutting that brittle fractures were main material removal regime for fused silica cutting. Irregular cross-sections of the cutting groove were always caused, as shown in Figure 7. The cross-sectional area *A* of the cutting groove for fused silica cannot be calculated by Equation (2). So the specific cutting energy was not discussed in this section. Further research is necessary to find the CUCT and to get the specific cutting energy for fused silica by using SCD tools with effective negative rake angle.

## 5. Conclusions

An experimental investigation with nanometric plunge-cutting test has been carried out on two hard-brittle materials of BK7 and fused silica. In the experiments, SCD tool with 0° rake angle was used under the same cutting speed of 300 mm/min. Cutting characteristics was studied with special concentration on the material-removal regime based on ductile-brittle transition. Based on the research, the following conclusions can be drawn:

(1) There existed a CUCT of 320 nm in nanometric cutting of BK7, which experienced ductile, ductile-brittle transition and brittle cutting stage successively with increasing undeformed chip thickness. Only brittle cutting was found for fused silica with the same cutting conditions when undeformed chip thickness was above 30 nm, and brittle fractures existed for its high hardness and low fracture toughness compared with BK7.
(2) Low surface roughness value could be obtained in ductile cutting and ductile-brittle transition cutting during BK7 nanometric plunge-cutting test. Brittle cutting process was confirmed to cause the significant increase of machined surface roughness irrespective of BK7 or fused silica. The key reason was attributed to the large amount of cracks and fractures on the machined surface.
(3) The feed cutting force almost increased linearly with elevated undeformed chip thickness for BK7, while the feed cutting force for fused silica fluctuates violently and the amplitude of fluctuation increases when undeformed chip thickness increased. The phenomena were mainly due to the large brittle fractures that led to the high cutting force and fluctuation.
(4) Size effect was found with undeformed chip thickness below 50 nm in ductile cutting of BK7. The appropriate undeformed chip thickness between 50 and 320 nm was expected to get a good surface integrity, when precision machining of BK7 with the similar cutting conditions as used in this research.
